# Hybrid Digital Twin Framework for Personalized Diabetes Management Using Mathematical Modelling and Machine Learning

**DOI:** 10.3390/diagnostics16172813

**Published:** 2026-09-01

**Authors:** Vathana Dennish, Babu Subramani, Vijayakumar Ponnusamy, Suganthi Kuppusamy, Janardhanan Subramonia Kumar, Nemanja Zdravković, Miloš Kostić

**Affiliations:** 1 Department of Computing Technologies, SRM Institute of Science and Technologies, Kattankulathur, Chennai 603203, India; vathanad@srmist.edu.in (V.D.); babus@srmist.edu.in (B.S.); 2 Department of ECE, Faculty of Engineering & Technology, SRM Institute of Science and Technology, Kattankulathur, Chennai 603203, India; suganthk@srmist.edu.in; 3 Department of General Medicine, SRM Medical College Hospital and Research Center, Kattankulathur, Chennai 603203, India; kumarj1@srmist.edu.in; 4 Faculty of Information Technology, Belgrade Metropolitan University, 11000 Belgrade, Serbia; nemanja.zdravkovic@metropolitan.ac.rs (N.Z.); milos.kostic@metropolitan.ac.rs (M.K.)

**Keywords:** explainable AI (XAI), Bergman minimal model (BMM), Bayesian risk estimation (BRE), digital twin (DT), hyperglycemia, hypoglycemia

## Abstract

**Background/Objectives**: Diabetes mellitus is a chronic metabolic disorder characterized by impaired regulation of blood glucose due to defects in insulin secretion, insulin action, or both. Physiological and lifestyle factors vary among individuals. General medicine is not applicable to all patients. In this scenario, personalized medicine for each individual becomes costly. Effective management of continuous glucose levels with accurate insulin dosage is challenging. To overcome this, a digital twin (DT)-based insulin dosage simulator with an individual’s metabolic system is proposed in this work. **Methods**: Various machine learning techniques, mathematical models of physiology, and risk assessment using probability are used to predict the dynamics of patient-specific glucose–insulin. Parameters such as carbohydrate intake, sleep patterns, medications, and physical activity were incorporated into this model to capture real-world variations in daily life. For glucose–insulin interactions, the Bergman Minimal Model (BMM) is used; for time-of-day variability, a circadian insulin sensitivity model is used; and for predicting metabolic risks, Bayesian risk estimation (BRE) is used, which includes hyperglycemia risk. To enhance transparency and interpret model predictions, explainable artificial intelligence (XAI) methods are employed. **Results**: The simulation results showed improved glucose prediction accuracy, enhanced detection of hypoglycemia risk, and optimized insulin dosing strategies compared with traditional approaches. **Conclusions**: Overall, the proposed digital twin model offers a scalable solution using the latest techniques A “Prescriptive Analytical Framework” is provided using the BMM and BRE for personalized diabetes management and decision support for clinicians.

## 1. Introduction

Diabetes mellitus is one of the most common chronic diseases worldwide, affecting millions of individuals. In the next few decades, the global burden is expected to increase substantially. Maintenance of blood glucose levels within a safe physiological limit is essential to prevent problems, from small acute complications to long-term damage to organs; it typically requires continuous monitoring [1], and this should be combined with insulin therapy for individuals.

Various multiple interacting factors that influence the regulation of glucose include one’s duration of sleep, dietary intake of carbohydrates, level of physical activity, psychological stress, adherence to medication, and circadian rhythmConventional treatment strategies rely on suggesting a generic insulin dosage based on generic guidelines and intermittent glucose measurements, which often fail to account for the variable effects of lifestyle and variability among patients. The concept of digital twins was introduced as a result of computational advances in recent years in healthcare, which assist in the virtual representation of physical systems that are continuously updated using real-world data. In the context of healthcare, the digital twin model is used to simulate a patient’s physiological responses and forecast the outcomes of different treatment strategies.

Designing a digital twin model of an insulin dosing simulator [2] is the main objective or aim of this research, which integrates machine learning-based prediction, explainable artificial intelligence, and physiological modelling. This system aims to provide decision support for patient-specific improved management of diabetes and to simulate personalized glucose–insulin dynamics [3].

## 2. Literature Review

To continuously monitor real-time clinical data, digital twins (DTs) facilitate predictive analysis, personalized treatment, and active intervention. The limitations of conventional static models are overcome by the transformative paradigm of creating a virtual replica, called a digital twin (DT) in the healthcare division. This also provides solutions using machine learning (ML) concepts for AI in the healthcare sector.

### 2.1. Digital Twin Frameworks for Diabetes

The literature survey addressed by Shamanna et al. [1] demonstrated a real-world DT intervention. Details on achieving significant improvements in glycemic control over a year were introduced in their paper. Zhang et al. [3] proposed a multi-layered DT framework incorporating ML, multi-omics, and knowledge graphs for precision diabetes care. These two scenarios lack real-time adaptive feedback mechanisms and the interpretability of clinical data. A comprehensive architecture for wearable sensors, electronic health records, and continuous glucose monitoring (CGM) is of utmost importance.

### 2.2. AI-Driven and Predictive Digital Twin Models

Despite the improvements made, DT models suffer from limited explainability, data dependency, and a lack of generalization at the outset. Arefeen and Faruqi introduced a DT system into the analytical framework. Arefeen et al. [4] introduced GlyTwin, which leverages counterfactual reasoning to optimize glucose control strategies. Faruqui et al. [5] proposed a nurse-in-the-loop DT system to improve the reliability of decision-making. Physiologically constrained neural networks (PCNNs) have also been explored to improve model realism, as explored by a group of scientists. Roquemen-Echeverri et al. [6] demonstrated improved glucose prediction accuracy by embedding physiological constraints into deep learning models.

### 2.3. Decision-Support Applications and Clinical Traits

Treatment optimization and risk prediction were the focus in clinical applications. Builes-Montaño et al. [7] developed a DT-based decision support system for insulin optimization, which improved treatment outcomes. Elias and König [8] modelled pharmacokinetics using DTs to personalize drug therapy.

Mishra et al. [9] incorporated system-level models based on policy and population-level simulations, enabling analysis, healthcare planning, and intervention in clinical data. These have major drawbacks that are evident in the survey report, including a lack of closed-loop control systems, IoT data streams cannot be integrated, and limited patient-specific explainability. Recent studies have demonstrated that diabetes management is shifting towards personalized and data-driven healthcare applications. AI-based DT models reveal real-world simulations and continuous monitoring of glucose levels.

From a survey of different papers analyzed, it is evident that recent DT models include clinical diabetic treatments (DTs) [1], AI-based DTs [4] hybrid nurse-in-the-loop models [5], physiologically constrained neural network DTs [6], and system-level policy DTs [9]. Most studies utilize real-world patient data, CGM measurements, or simulated datasets combined with machine learning, knowledge graphs, or system dynamics modelling.

Continuous monitoring [10] significantly improves glycemic regulation, enhances glucose prediction capability, and supports more effective insulin dose adjustments. Integrated DT frameworks that combine physiological modelling with adaptive computational methods are being developed. The DT provides clinician-guided decision support [11] with improved personalization and operational reliability. Furthermore, advanced modelling strategies, including causal inference methods and physics-guided computational models, are required. A mathematical optimization [12] approach enables more accurate and stable glucose management. Comprehensive system architectures that incorporate multi-omics information and electronic health records facilitate a broader understanding of patient-specific metabolic behaviour. In addition, pharmacological [9] and large-scale system DTs expand the applicability of these frameworks toward medication response analysis and healthcare management [13] planning.

Despite these advancements, several challenges remain unresolved. High computational requirements, difficulties in large-scale deployment [14], reduced model interpretability, and limited real-time adaptability continue to affect their practical implementation. Moreover, many review-based studies primarily focus on theoretical developments and future research opportunities without sufficient experimental validation or real-world clinical deployment [15] These limitations highlight the growing need for scalable, interpretable, and patient-centric DT systems capable of supporting reliable and personalized diabetes management.

## 3. Related Work

In this section, we review prior research on physiological modelling in glucose–insulin, glucose prediction by machine learning approaches, and the application of digital twins in healthcare.

### 3.1. Physiological Glucose–Insulin Models

To describe the interaction between insulin and glucose within the human body using mechanistic equations and physiological models, the Bergman Minimal Model [10] was used, which captures key processes such as insulin-mediated uptake of glucose and glucose production by hepatic cells. In artificial pancreas research, the Hovorka Model [16] is employed and frequently adopted; it includes all sub-models in detail for the absorption of insulin, metabolic utilization, and distribution of glucose across various compartments.

Although the proposed model provides good interpretability of physiological characteristics, it also has limitations. It requires the identification of complex parameters and has difficulty for incorporating patient-specific lifestyle factors, including dietary habits, exercise patterns, and sleep behavior, which limits its real-world applicability for personalization.

### 3.2. Machine Learning Models for Glucose Prediction

Machine learning techniques are used extensively to predict glucose levels by using data from continuous glucose monitoring (CGM) systems [4] and their related clinical variables. Commonly used methods include Support Vector Machines (SVM), Long Short-Term Memory (LSTM) [17] recurrent neural networks, and feedforward neural networks. By learning temporal patterns and nonlinear relationships from large datasets, these models achieve the highest predictive performance for both short- and medium-term glucose forecasting. However, purely data-driven approaches typically suffer from limited interpretability of physiological properties. Although they can provide accurate numerical predictions, they do not represent the underlying biological mechanisms of glucose–insulin regulation, which makes it difficult for clinicians to understand and trust the basis of the outputs of this model.

### 3.3. Digital Twin Approaches and the Proposed System Novelty

Existing diabetes management systems generally rely on isolated physiological models or machine learning techniques and often lack individualized adaptation, uncertainty estimation, explainability, and integration of lifestyle and circadian factors. The main aim of this digital twin framework is to combine the mechanistic physiological model with data-driven analytics to construct a virtual representation for personalization that is tailored to individual patients. These virtual models enable clinicians to conduct in silico experiments and evaluate alternative treatment strategies, and the potential risks must be assessed before applying the interventions in regular practice.

Many existing digital twin implementations [3,4,5,6,7,10,16] for metabolic diseases do not fully integrate all key aspects, such as lifestyle behaviour modelling, circadian metabolic variability, and explainable AI [17,18,19] components. The present work on digital twins addresses these gaps by incorporating the probabilistic estimation of risk, circadian rhythm modelling [6], and XAI within the insulin dosing framework of digital twins. This study focuses on personalized medicine instead of generalized medicine. This invokes the trend for technological change, which is a futuristic goal. The BMM estimates patient-specific insulin and glucose effectiveness. Hovorka Model will simulate detailed glucose–insulin dynamics, including meal and insulin effects. By combining these two models, personalized insulin dosage, accurate prediction of glucose, and adaptive diabetes management were supported by our DT framework.

**Figure 1:**
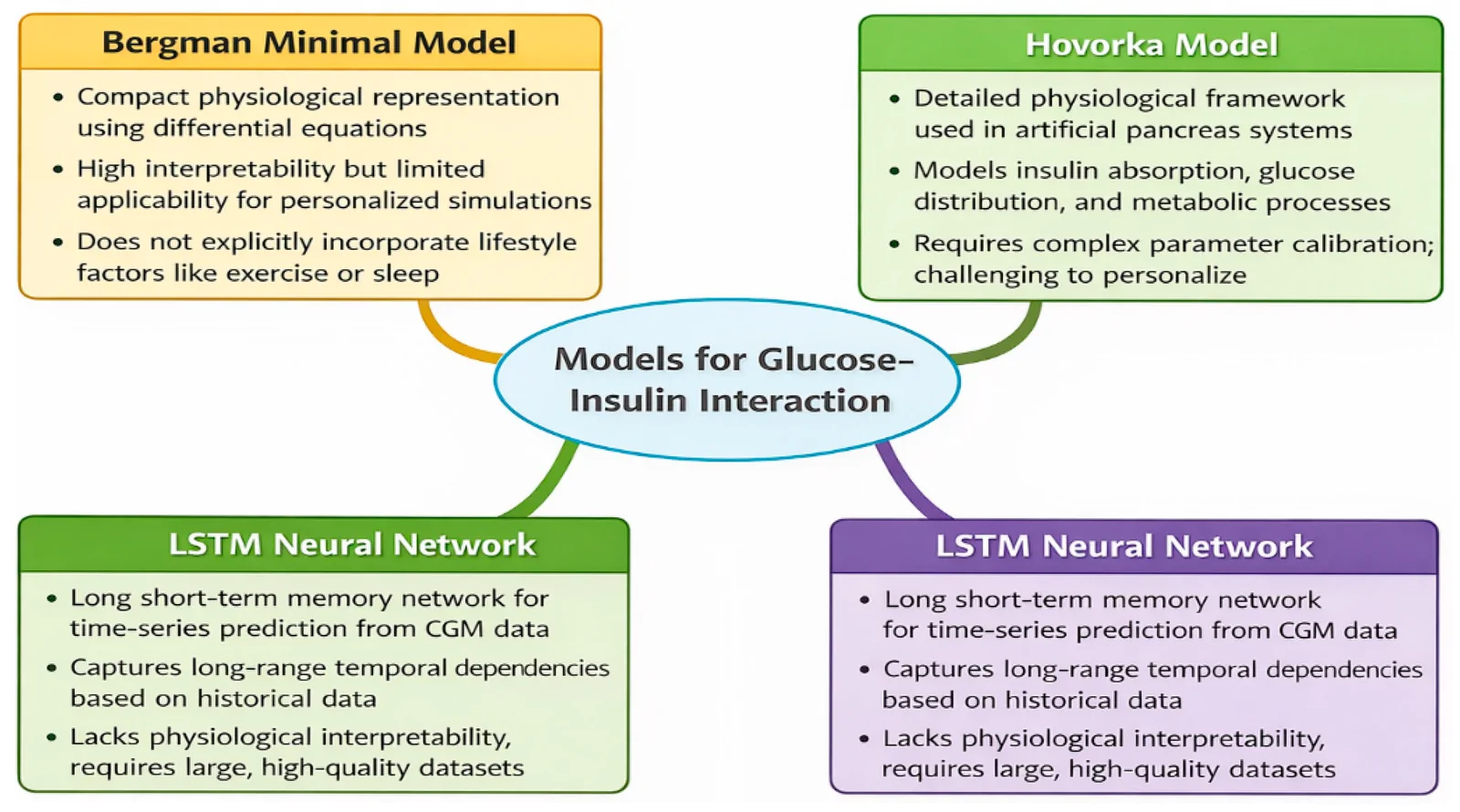
Existing machine learning-based glucose prediction models.

Table 1 presents a comparison between the different identified models. In addition, Figure 1 demonstrates the features of these ML- and DL-based algorithms [18,19] for developing the models.

In Figure 1, the existing machine learning-based glucose prediction models are presented with their salient features. From the literature survey, research gaps were identified. The proposed system is described in the following section.

Table 2 presents the research contributions of this study. Collectively, these contributions advance accurate and trustworthy personalized diabetes management in a smart, interpretable, and efficient risk-aware DT model suitable for clinicians and patients.

## 4. Proposed System Architecture

**Figure 2:**
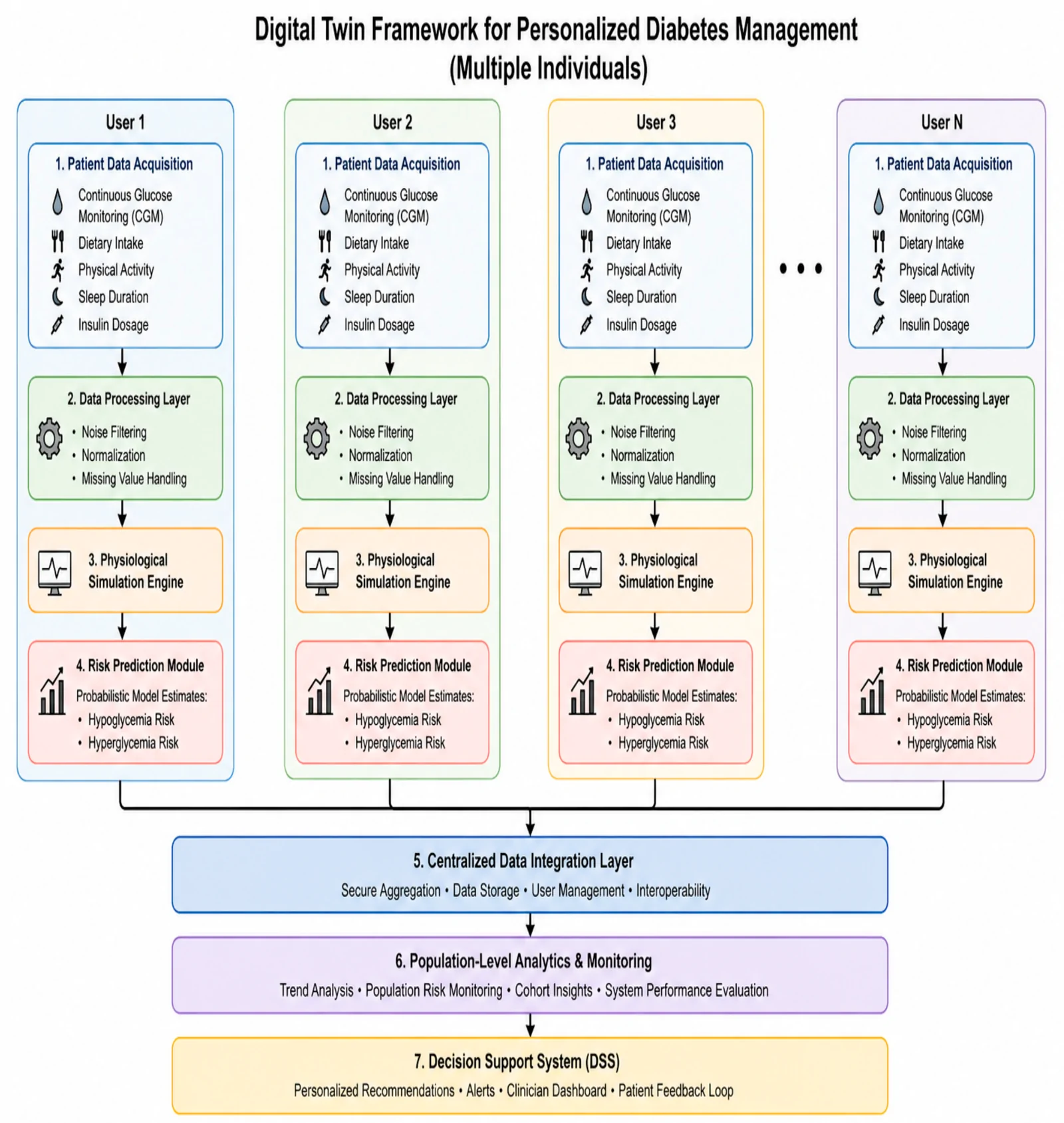
Block diagram of the DT framework for personalized medicine.

There are various interconnected modules in the proposed DT system that collectively simulate the metabolic behaviour of the patient and support the insulin dosage recommendation decision. Figure 2 demonstrates the DT framework [10,13] for personalized diabetes management for ‘n’ users. The following methods are proposed: patient data acquisition, data processing layer, physiological simulation engine, risk prediction model, centralized data integration model, population-level analytics, and decision support system (DSS). The individual blocks are elaborated in detail.

### 4.1. Patient Data Acquisition

Lifestyle-related data and physiological data are collected by web or mobile applications at regular time periods from the patient. This can be used as a data acquisition module for personalized medicine. Continuous glucose monitoring (CGM) [6] measurements are the primary input, and other primary inputs include information on dietary intake, with an emphasis on the carbohydrate content, levels and timing of physical activity, duration and schedule of the sleep cycle, and historical records on insulin dosage. The time-dependent description of the patient’s metabolic states and daily routine works are comprehensively provided by the collected data. As per the block description shown in Figure 2, the data acquisition part is carried out for ‘*n*’ users.

### 4.2. Data Processing Layer

Measurement artefacts, sensor noise, and missing values are some of the issues that often affect the quality of raw data. Data preprocessing [13,15] is performed in the processing layer to enhance data reliability and quality. To mitigate sensor fluctuations, noise filtering must be performed, which will bring different variables into comparable values in numerical scale normalization to handle incomplete or missing entries. Imputation strategies must be performed. These steps ensure that the prediction modules and downstream simulations operate on robust and consistent data.

### 4.3. Physiological Simulation Engine

In the DT model, the core is a physiological simulation. Multiple mathematical sub-models are integrated to represent the essential processes of metabolism, including the absorption of glucose from meals, the action and secretion of insulin, the effect of physical activity on the utilization of glucose, and the variations in circadian metabolic sensitivity. The simulation engine generates individualized, time-resolved representations of the glucose–insulin dynamics [5,6] of the patient under varying treatment inputs and lifestyle conditions by combining these components for user ‘1’ to user ‘n.’

### 4.4. Risk Prediction Module

By using probabilistic models, with reference to Section 5 and convergence towards Equations (1)–(3), the occurrence of adverse metabolic events is revealed, which is evaluated by the risk prediction module. Based on the predicted and current metabolic states, the probabilities of hyperglycemia (excessively high blood glucose) and hypoglycemia (dangerously low blood glucose) were computed. To prevent acute complications, early estimation of these risk probabilities is helpful.

### 4.5. Decision Support System

The outputs of the simulation and risk prediction modules to derive recommendations on insulin dosage were interpreted by the decision support system. This aims to maintain regular blood glucose levels within a safe predefined range, and the estimated level of risk and predicted glucose trajectories will be considered. This module aims to aid healthcare professionals in tailoring insulin therapy to individual patients by functioning as a decision support tool for clinical purposes. As per the block description in Figure 1, this task is accomplished by a dashboard and web-based application, which leads to automation. Personalized medicine [2,3,4,5,6,7,8,9,10,20,21,22,23,24,25,26,27,28,29] can be administered to individuals by continuous monitoring of clinical traits.

## 5. Mathematical Modelling

The following mathematical model is employed in the important physiological process underlying insulin and glucose regulation [6,7]. This computational basis for the DT model is used to simulate how the blood glucose level responds to various inputs, such as physical activity, food intake, sleep cycle, and insulin administration.

As active physiological models [12,14], the simulators deploy mathematical models, such as the Bergman Minimal Model for glucose–insulin dynamics, a Bayesian model for risk assessments, and circadian sensitivity adjustments. All simulations were performed for the above-mentioned models, which were run in real time using JavaScript Version ES2026.

Personalized Parameters:

The constants α, β, γ, θ, λ, and δ are personalized parameters that vary between individuals based on physiological characteristics such as the following:

- Age;
- Body mass index (BMI);
- Insulin sensitivity;
- Metabolic rate;
- Medical history;
- Gender.

Personalizing these parameters allows the DT to replicate the unique metabolic behaviour of each patient, thereby improving prediction accuracy and treatment planning.

### 5.1. Mathematical Model

**Figure 3:**
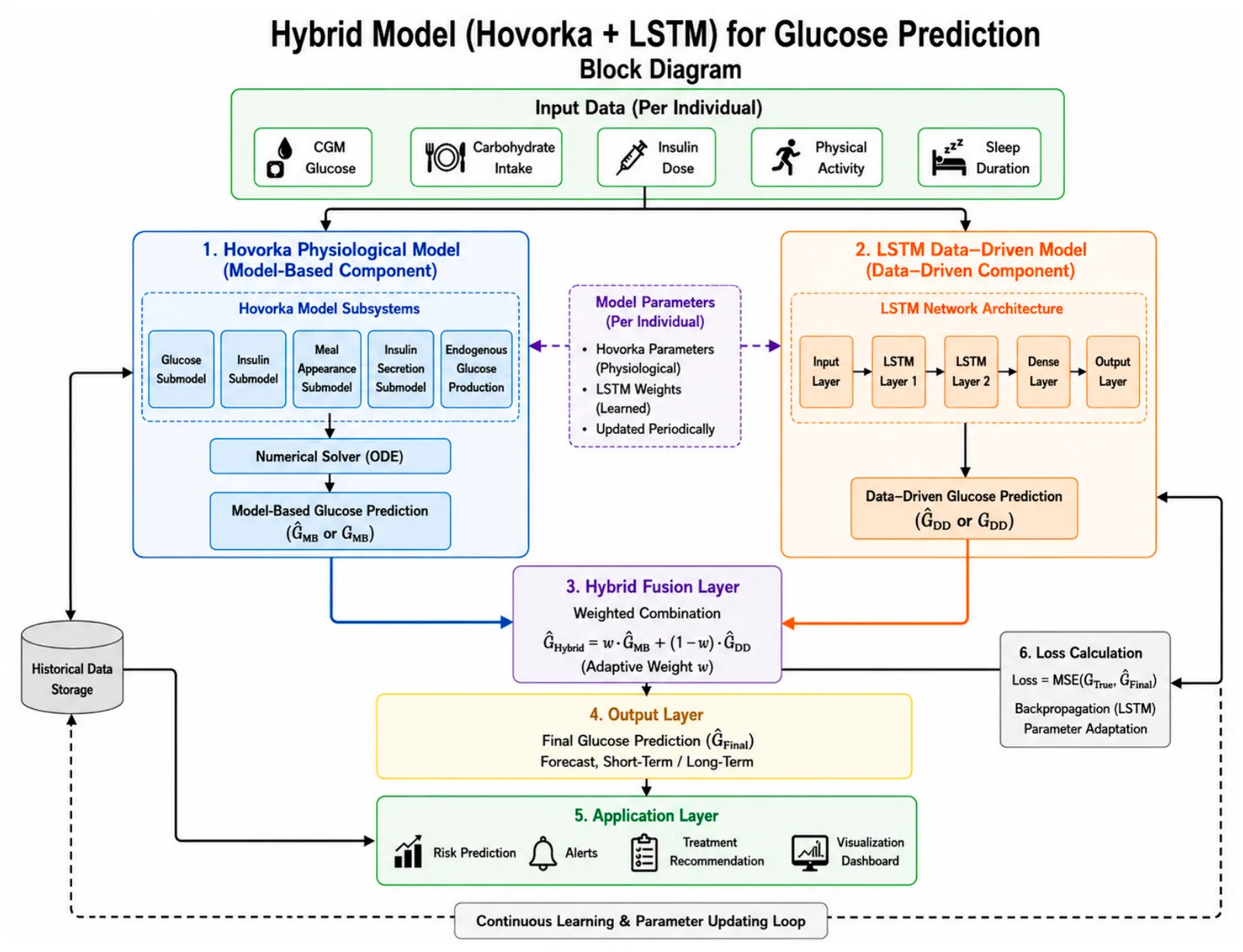
Proposed model.

The proposed DT model, as shown in Figure 3, has a dynamic relationship between the blood glucose level and insulin regulation by using simplified mathematical equations. The equations simulate how changes in different lifestyle factors, such as food intake, physical activity, medication, and sleep patterns, influence the metabolism of glucose in the human body.

Representing the glucose and insulin dynamics over time, this model consists of Equations (1) and (3), given below with a detailed description.

#### 5.1.1. Glucose Regulation Equation

The predicted blood glucose is given by:

(1) $$G(t+1)=G(t)+\alpha [F\left(t\right)]-\beta [A\left(t\right)]-\gamma [I(t)]+\delta [8-S\left(t\right)]$$

Glucose level refers to the current level of glucose in the blood. The blood glucose level at the next time step is predicted by Equation (1), which is based on the current physiological state and lifestyle inputs. The typical range of TG is 70–300 mg/dL. Using a continuous glucose monitor (CGM) or Finger-Stick Glucometer, it is estimated and calibrated for each individual CGM.

where

G(t) represents the blood glucose level at time t, measured in mg/dL.

G(t + 1) represents the level of blood glucose predicted at the next time step, additionally considering metabolic influences.

Glucose dynamics are influenced by the parameters listed below.

#### 5.1.2. Food Intake (α[F(t)])

The blood glucose level increases significantly with the consumption of carbohydrates. The rate at which dietary carbohydrates are converted into glucose in the bloodstream is represented by parameter **α**. A larger increase in glucose concentration occurs as a result of a higher carbohydrate intake. The numerical model describing the conversion of food to carbohydrates can be estimated using food diaries and mobile nutrition apps, with a range of 0–150 g/meal. Glycemic index adjustment is a personalized method used in this study. Carbohydrate consumption, through intestinal absorption, increases the glucose concentration.

#### 5.1.3. Physical Activity (β[A(t)])

Muscles utilize glucose as an energy source, which lowers blood glucose levels during physical activity. The effectiveness of physical activity in reducing blood glucose concentration is represented by parameter β. The value of this parameter can be analyzed before and after physical activity, which varies among individuals. This is presented in detail in Section 5.1. Glucose energy dynamics are modeled using the well-defined rule-based model proposed by Hovorka, together with an LSTM model. The physical activity parameter β varies between individuals: it represents the expenditure of energy by performing exercise; it enhances the uptake of glucose by the muscles of the skeletal system; and it lowers the glucose in the blood. The range was 0–10 MET·h/day, with kcal used as the unit of measurement. Smartwatches and individual fitness trackers can be used for estimation. For the personalization method, individual activity is tracked.

#### 5.1.4. Insulin Effect (γ[I(t)])

Insulin plays a crucial role in the transport of glucose from the bloodstream into cells. Insulin sensitivity, which is how effectively the level of glucose is reduced by insulin, is represented by parameter **γ**. High insulin levels lead to a greater uptake of glucose by body tissue. Individual 1 represents a single person (n1). I(t) comprises internal (insulin effects within the body) and external (due to external physical activity) parameters, as given by Equation (2). The range is 0–20 U, the fitness tracker is the estimation method, and the personalization method is based on the patient-specific insulin sensitivity factor (ISF) and insulin action profile.

I(t)_n1_ = I(t)_int_ + Int_ext_ (2)

#### 5.1.5. Sleep Influence (δ[8−S(t)])

Metabolic regulation is affected by sleep duration and quality. Increased glucose levels are observed in people with insufficient sleep, which can impair insulin sensitivity. In this proposed model, 8 h of sleep is considered an optimal duration. If sleep is less than 8 h, the term (8-Sleep) becomes positive, indicating an increase in glucose levels due to metabolic stress. The magnitude of this effect is determined by parameter **δ**. Daily lifestyle factors influence fluctuations in glucose levels in the human body. This parameter can be recorded for each day in a week for a trial of 3 months’ duration. Thus, we can interpret sleep influence data. The duration and timing of sleep influence the modulation of the metabolic rate and insulin sensitivity. Personalized baseline sleep duration is estimated with help of a sleep tracker.

The range values of coefficients are: α is (1–5) mg/dL per g, β is (2–10) mg/dL per MET·h, γ is (20–80) mg/dL per U, and 8 h sleep is mg/dL per hour sleep deficit.

#### 5.1.6. Insulin Regulation

Circulating insulin facilitates cellular uptake of glucose and suppresses its production by hepatic cells. This is governed by pancreatic insulin secretion function and analysis of blood glucose levels. When the blood glucose level is high, the pancreas releases insulin. The strength of the response, which represents pancreatic efficiency, is controlled by functional parameters. Insulin injections or glucose-lowering drugs are often used by patients with diabetes. Greater insulin availability in the blood was due to a higher dosage of medication. The following Equation (3) allows the DT to simulate both the medically assisted regulation of insulin and the natural production of insulin.

(3) I(t+1)=I(t)+θ⋅f(G)−(λ⋅Medication)

The change in the concentration of insulin over time is represented by the proposed model with the above Equation (3), which considers both external medications and secretion of the pancreas.

where

I(t) represents the level of insulin at time t, measured in mU/L.

I(t + 1) represents the level of insulin at the next time step.

f(G) is the physiological response of the pancreas to glucose metabolism.

θ represents the strength of the response, which represents the efficiency of the pancreas.

(θ f(G)) represents pancreatic insulin secretion.

λ is the effectiveness of the medication in altering the insulin level.

(λ Medication) represents Medication Influence.

The nonlinear relationship between the glucose concentration and insulin secretion by beta cells of the pancreas is given by the function f(G). In healthy human physiology, the secretion of insulin gradually increases with a rise in glucose level and reaches saturation at very high concentrations of glucose. A typical form of this function can be expressed by Equation (4):

(4) $$f(G)=\frac{G}{G+K}$$

where K is a constant (sensitivity constant) that determines how fast the secreted insulin responds to increasing glucose levels. When glucose levels are low, insulin secretion remains minimal. As the glucose level rises, the rate of secretion increases until a physiological limit is reached. Where G is the glucose level indication.

### 5.2. Glucose Dynamics Model

The temporal evolution of the glucose concentration in the blood is described by the glucose dynamics model. The level of glucose is influenced by various factors as per the formulation in Equation (1): glucose concentration, food intake, physical activity, insulin concentration, and sleep factor. By combining these effects, Equation (1) is updated at each time step in the simulation. The DT model can reproduce realistic fluctuations in glucose levels over the daily living time, which is enabled by this simulation time interval.

### 5.3. Insulin Regulation Model

The concentration changes in insulin in the human body over time are characterized by the model of insulin regulation, as given in Equation (3). The two primary components for analysis are exogenous administration of insulin, accomplished by insulin delivery via pumps or injections, and endogenous pancreatic secretion of insulin, which is based on the response to elevated blood glucose levels and insulin produced by the β-cells in the pancreas. The secretion of insulin by the pancreas is modelled using a nonlinear function of glucose, by capturing the biological behaviour in observation, whereby once glucose in blood exceeds a certain threshold value, the release of insulin rapidly increases. This combined model determines the amount of insulin needed to regulate blood glucose levels and drive glucose uptake by the cell.

### 5.4. Circadian Sensitivity Model

The metabolic processes in humans are governed by circadian rhythms, which follow an approximately 24 h cycle. The sensitivity of insulin varies throughout the day, typically being higher in the morning and gradually decreasing during the late evening. The sensitivity model of the circadian system incorporates a time-dependent factor into the equation of glucose dynamics, which is used to represent daily changes.

By adjusting the sensitivity of insulin as a function of time over a day, this model improves the accuracy and realism of the predicted response of glucose, particularly in full-day profiles or when simulating them on a long-term basis.

C(t) = Asin (2π/24(t − ϕ)) + B(5)

where

C(t) represents circadian sensitivity at time t.

A represents the amplitude of the rhythm.

B represents baseline sensitivity.

t represents time in hours.

ϕ represents the phase shift.

### 5.5. Bayesian Risk Estimation—Equation Required

The probability of risks due to metabolic effects, such as hyperglycemia and, in some cases, hypoglycemia, is estimated using Bayesian inference. Prior knowledge about the level of risk from the observational data, which is incoming from the DT, and measurements from patients, is combined by the Bayesian model. Now, new data are available, and the estimates of probability are updated sequentially. These probabilities of identifying risk are used to generate alerts to the user, which indicate potential future glycemic excursions and support decisions related to insulin dosage at a safer level by quantifying risk and uncertainty. The probabilistic equations are given by the following equation:

P(R∣E) = P(E∣R)P(R)/P(E)(6)

## 6. Proposed Digital Twin Model—Working Principle

**Figure 4:**
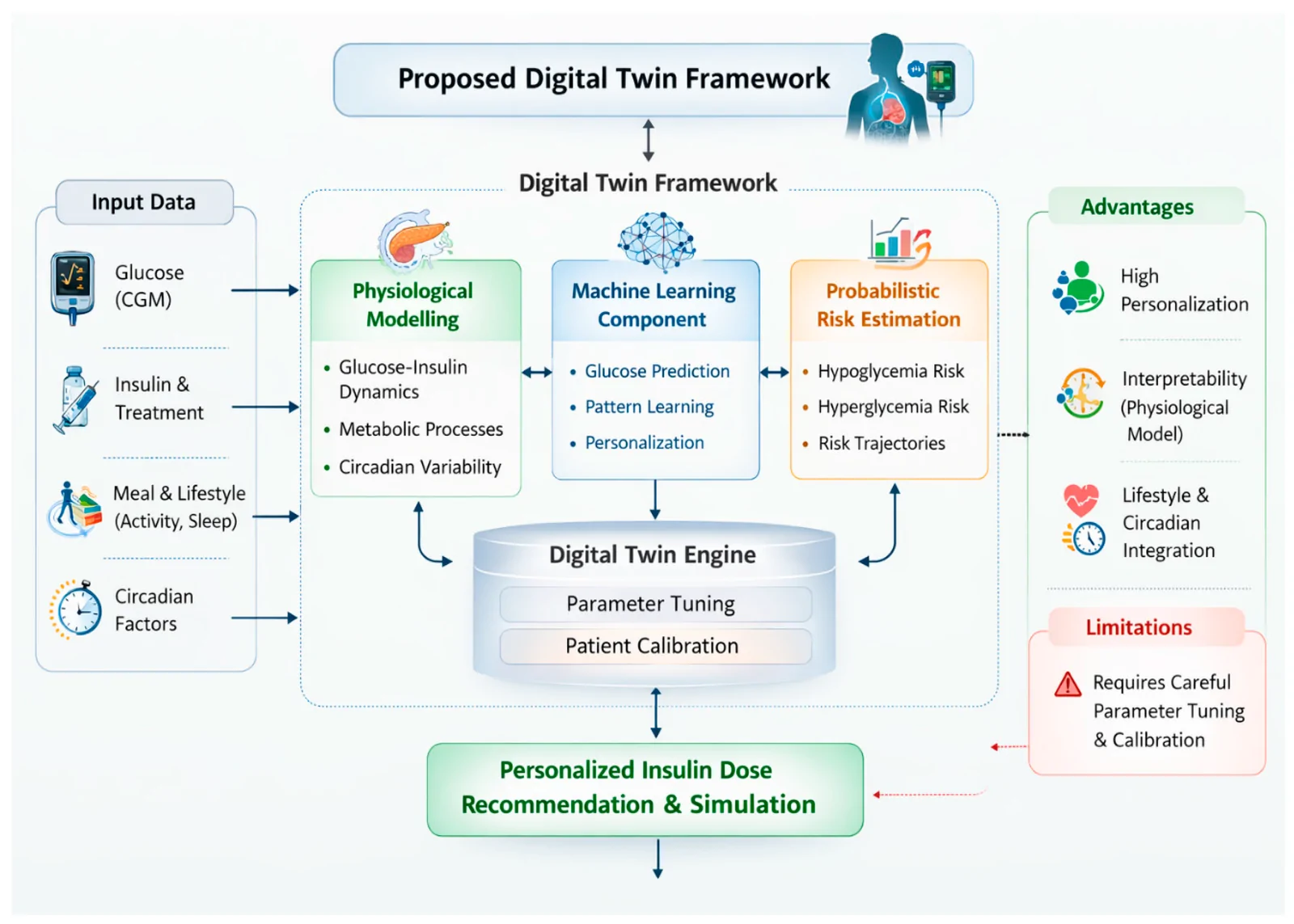
DT framework—AI based.

The proposed DT model, shown in Figure 4, combines the machine learning components, physiological modelling, and estimation of risk in a probabilistic manner within the framework of a unified digital twin. The main advantages of this model include improved interpretability owing to the principle of the underlying physiological structure, high personalization, and explicit integration of various factors related to lifestyle and variability due to circadian cycle activity. A very important factor, which is considered to be the key limitation of the proposed DT model, is that it must be carefully calibrated for each patient, and careful tuning of the parameters must be performed.

The DT model [15,20] are functioning by continuously simulating the interactions of metabolic actions between insulin and glucose in response to real-world inputs. When a patient consumes a meal that is rich in carbohydrates, their glucose level in the bloodstream increases. This sudden increase stimulates the release of insulin from the pancreas, which facilitates the uptake of glucose by body tissues, such as fat cells and muscles. Muscles utilize glucose during physical activity, which helps reduce blood glucose levels. Adequate sleep of approximately 8 h supports proper metabolic activity, which improves insulin sensitivity. Conversely, insufficient sleep can impair metabolic regulation and activity, leading to high glucose levels. Medications such as glucose-lowering drugs and insulin injections contribute to maintaining the balance of glucose. Combining these factors with mathematical models, the DT model simulates real-time changes in metabolic activity and predicts the future level of glucose. These predictions can assist in determining the appropriate insulin dosing strategies tailored to each patient’s lifestyle and physiological conditions.

### 6.1. Data Collection and Experimental Setup

Particulars of volunteers, such as name, age, sex, details of the meal taken, the time between the reading and a meal, whether an individual had diabetes, if the volunteer was on any medications, physical activity in daily life, height, weight, sleep status, stress, SP02, hair on the wrist, and any other health complications, were recorded [28]. The volunteer’s finger was pricked with a lancet. A non-invasive method of obtaining blood glucose values from the hardware was used. Wrist wearables were tied to the wrist of the volunteer to obtain blood glucose values [28]. The extension of the work with 201 volunteers was carried out here with the physically collected dataset. The niGLUC-2.0v device was calibrated using glucose readings from a clinically validated reference glucometer. Linear regression was used for calibration, and three consecutive readings were obtained for each participant; the average value was used for analysis. MAE, RMSE, and MAPE were used to evaluate device performance. The non-invasive monitoring niGLUC 2.0v device provides accurate and reliable measurements.

**Figure 5:**
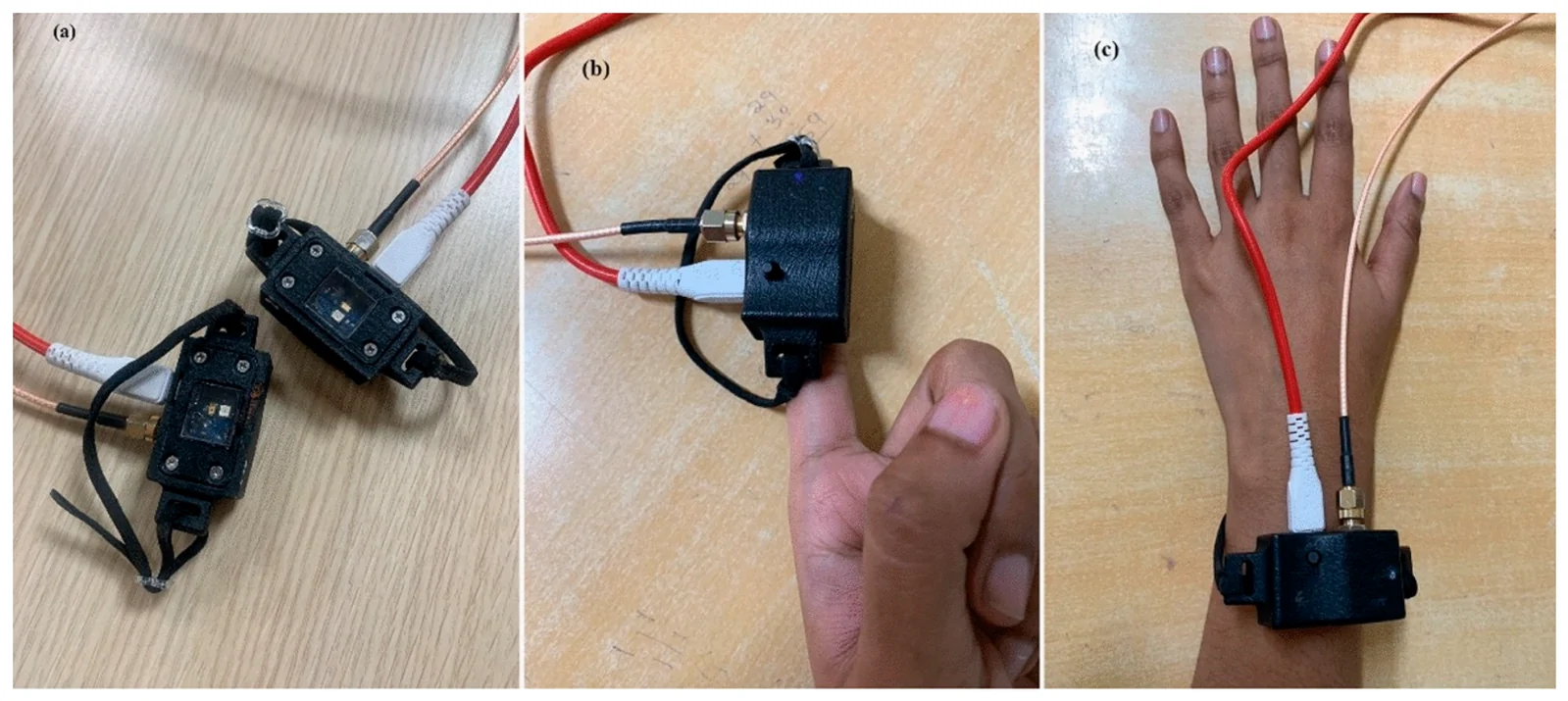
The hardware setup of niGLUC-2.0v [28]: (**a**) finger and wrist sensors; (**b**) measurements from the finger sensor; (**c**) measurements from the wrist sensor.

The performance of the proposed DT system must be evaluated using individual patient profiles. Simulation experiments [28] were conducted in our previous work, and they represent the typical metabolic behaviour under regular living conditions in day-to-day activities. The hardware prototype designed in a previous study [28] is shown in Figure 5. The simulation had a sampling interval of 6 min and was performed over a period of 24 h. The three main meal events and two (structured) physical activity sessions reflect the realistic pattern of a regular lifestyle. These proposed settings enable the assessment of the ability of a system to handle meal-induced excursions of glucose and exercise-related reduction in blood glucose within a full-day context.

### 6.2. Proposed Method—Flow Diagram

**Figure 6:**
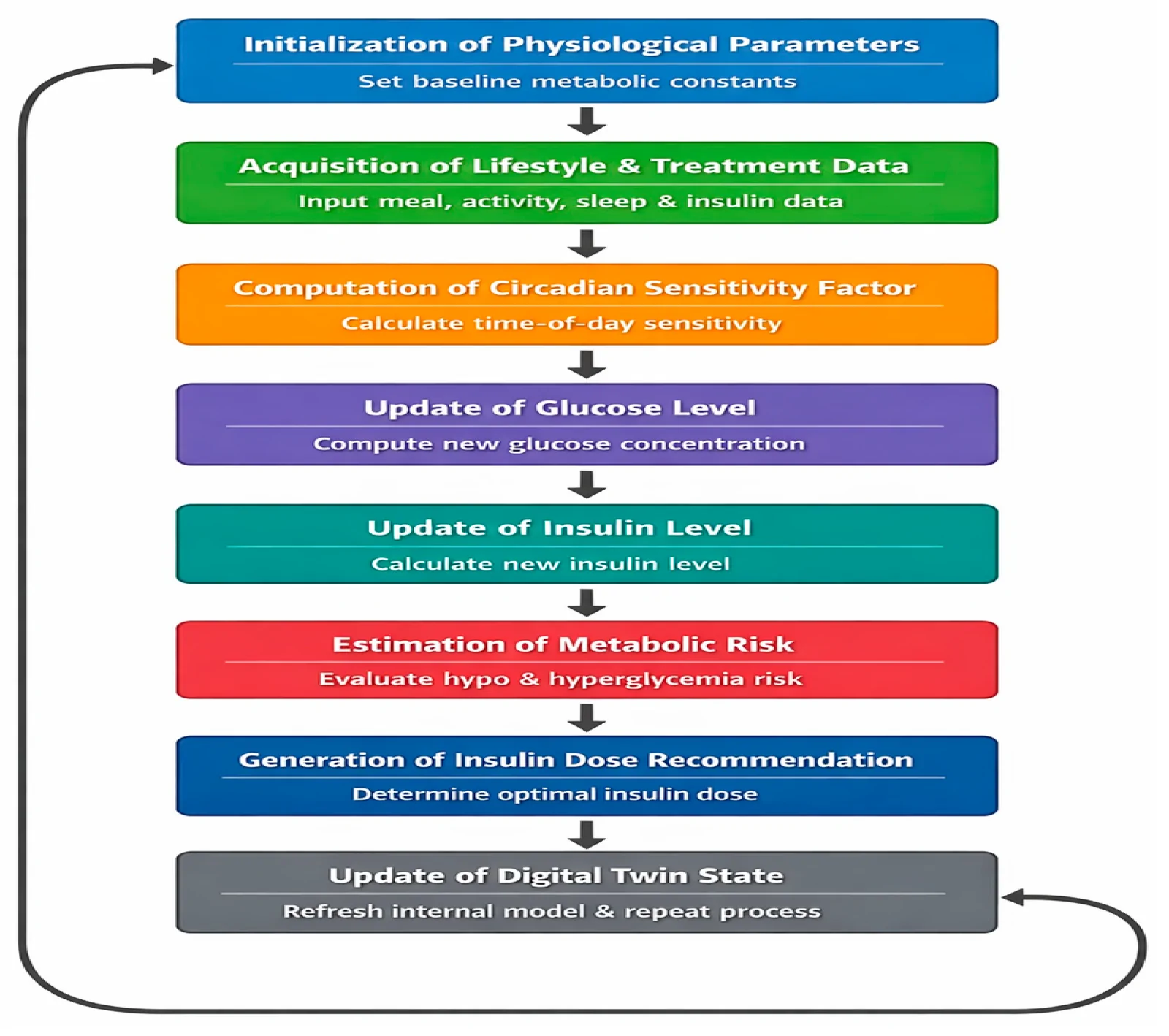
Flow chart of metabolism simulation.

The proposed algorithm of the DT simulator defines the procedure in a stepwise manner to predict the profile of glucose and recommend the dosage of insulin. The methodology to be followed is shown in Figure 6. This system initializes the specific parameters, which are the key features of the patient, including the sensitivity of glucose, response rates of insulin, and other constants of metabolic processes. The individual’s baseline metabolic characteristics are encapsulated by specific parameters, as per Equations (1)–(6).

Real-time data on the intake of meals rich in carbohydrates, as well as the duration and intensity of physical activity, duration of sleep, and insulin dosage history were obtained. All these variables served as inputs to the simulation. The parameters α, β, γ, θ, λ, and δ were collected from individuals through mobile and web-based applications. The computation of the circadian sensitivity factor is based on the current time of day that modulates the sensitivity of insulin and the response of metabolism in the physiological models, as given in Equations (1)–(5).

The simulator computed the new concentration of glucose by using the dynamics model of glucose by incorporating the effects of food intake, the current level of insulin, modulation of the circadian cycle, and physical activity with respect to Equation (1). The exogenous administration of insulin was combined with the endogenous secretion of pancreatic insulin, and it was updated in the insulin regulation model in response to the updated level of glucose, as per Equation (3). The probabilities of occurrence of hypoglycemiaand hyperglycemia were evaluated using the Bayesian risk estimation module, which is based on the predicted trajectories and updated state of metabolism. The system determines an optimal insulin dose, which is needed to maintain the glucose within a target range while ensuring risk minimization by considering both estimated risks and the predicted level of glucose. To reflect the new condition of the metabolic state, the internal state of the DT model was updated, and this procedure was repeated continuously to enable the real-time simulation of factors. The algorithm below is used for the evaluation of the DT-based design.

The inference from the clinically validated reference glucometer, the niGLUC-2.0v device, was calibrated using glucose readings. The niGLUC 2.0v device provides accurate and reliable measurements. Table 3a,b provide the details of the dataset used along with the samples. The 24 h digital twin simulation is driven using clinically acquired patient data collected throughout the monitoring period. The simulation reproduces patient-specific glucose–insulin dynamics using measured physiological inputs rather than synthetic data alone.

Algorithm 1 provides the step by step process of the proposed hybrid DT for personalized glucose prediction.
**Algorithm 1.** Hybrid DT for Personalized Glucose Prediction        **Input:** Data $D=\{G_{t},I_{t},F_{t},A_{t},S_{t},M_{t}\}$ $G_{t}-Glucose\;intake$ $I_{t}-Insulin\;level$ $F_{t}-Food\;intake$ $A_{t}-Physical\;Activity$ $S_{t}-Sleep\;factor$ $M_{t}-Medication$        **Step 1: Normalize glucose dynamics to enable consistent temporal learning.**$G_{t}^{norm}=\frac{G_{t}-G_{min}}{G_{max}-G_{min}}$(7)        **Step 2: Model glucose evolution using the proposed lifestyle-aware DT formulation.**
$G_{t+1}=G_{t}+\alpha F_{t}-\beta A_{t}-\gamma I_{t}+\delta (8-S_{t})$(8)        The estimation constants α, β, γ, and δ are computed based on the mathematical model formulation in equations 7 and 8.        **Step 3: Compute physiological glucose–insulin interaction using the Bergman minimal model.**$\frac{dG}{dt}=-(X+p_{1})(G-G_{b})+D_{t}$(9)$\frac{dX}{dt}=-p_{2}X+p_{3}(I-I_{b})$(10)where p1 to p3 are the probabilitiesused for prediction in the models.        **Step 4: Simulate metabolic regulation using the Hovorka nonlinear model.**$\frac{dG}{dt}=-1-X_{1}G+kF_{t}$(11)$\frac{dX_{1}}{dt}=-aX_{1}+bI_{t}$(12)        **Step 5: Temporal dependencies are captured using LSTM-based sequence modelling for prediction.**
$X_{t}=[G_{t-10},\ldots ,G_{t-1}]$(13)${\hat{G}}_{t}=f_{LSTM}(X_{t})$(14)        **Step 6: Quantify prediction accuracy using statistical performance metrics.**
$RMSE=\sqrt{\frac{1}{n}\sum (G_{t}-{\hat{G}}_{t}{)}^{2}}$(15)$MAE=\frac{1}{n}\sum |G_{t}-{\hat{G}}_{t}|$(16)$R^{2}=1-\frac{\sum (G_{t}-{\hat{G}}_{t}{)}^{2}}{\sum (G_{t}-\bar{G}{)}^{2}}$(17)        **Step 7: Interpret model behaviour using SHAP-based feature attributions.**
$\hat{G}=f(F,A,S,M)$(18)$\phi_{i}=\mathrm{Contribution}\;\mathrm{of}\;\mathrm{feature}\;i$        For i = 1 to n, iterations are performed.        **Step 8: Assess system stability through eigenvalue analysis.**Stability(t) = f(λi, ω, ζ, Et)(19)where λi—eigenvalues; ω—natural frequency; ζ—damping ratio; Et—environmental/system conditions.        **Step 9: Validate asymptotic behaviour using Lyapunov stability criteria.**
$V(x)=x^{T}Px$(20)$\dot{V}=A^{T}P+PA$(21)        $V\left(x\right)$ represents **the deviations of glucose and insulin from their physiological equilibrium states.**        x represents the system state variables,$x=[G_{t}-G^{*},\;I_{t}-I^{*}{]}^{T}$(22)where $G_{t}$ is the current glucose value, $I_{t}$ is the current insulin value, and $G^{*},I^{*}$ are equilibrium (target) values.        V(x) **is the Lyapunov Function— energy function that** measures system stability.        If V(x)>0, the system has a deviation.        $If\;V\left(x\right)=0$, the system converges to equilibrium.$Choose\;P=\left[\begin{array}{cc} p_{1} & 0\\ 0 & p_{2} \end{array}\right]\;\;$(23)$V(x)=p_{1}(G_{t}-G^{*}{)}^{2}+p_{2}(I_{t}-I^{*}{)}^{2}$(24)        Stability exists if V(x) < 0.        **Step 10: Estimate the clinical risk using probabilistic modelling:**        $P\left(\mathrm{Hyper}\right)=1-\Phi \left(\frac{180-\mu }{\sigma }\right)\;\;\;\;$(**Probability of Hyperglycemia for**
G>180 mg/dL);        $P\left(\mathrm{Hypo}\right)=\Phi \left(\frac{70-\mu }{\sigma }\right)$(**Probability of Hypoglycemia for**
G<70 mg/dL); where Φ(z) **is the Standard Normal Cumulative Distribution Function (CDF).**Φ(z)=P(Z≤z)(25)$\mu =\frac{1}{n}\sum G_{t}$(26)$\sigma =\sqrt{\frac{1}{n}\sum (G_{t}-\mu {)}^{2}}$(27)        **Step 11: Generate analytical outputs for evaluation and interpretation.**
${\hat{G}}_{t},\;RMSE,\;MAE,\;R^{2},\;\mathrm{Explainability},\;\mathrm{Stability},\;\mathrm{Risk}$$where\;RMSE,\;MAE,\;and\;R^{2}$ are used for prediction performance measure.        Explainability provides suggestions related to insulin intake.        Risk denotes the probability of predicting risk at earlier stages.        **Step 12: Rule-based recommendations for insulin dosage, medication, physical activity, sleep, and food nutrition.**

Personalized medicine with clinical traits was suggested for individuals. The algorithmic steps 1–12 are correlated with the mathematical Equations (1)–(24). Section 6.3 reveals that the algorithmic approach validates the simulation results.

### 6.3. Interpretation and Visualization

The DT model provides a graphical visualization that helps users understand the simulation results of insulin and glucose dynamics.

The current glucose and insulin levels are displayed by a bar graph, which allows users to easily observe the metabolic trends over time.

Additionally, this DT system visualizes the secretion of pancreatic insulin using animated bubbles positioned above the graphical representation of the pancreas. The bubbles indicate the insulin secretion intensity.

1. Blue bubbles indicate low secretion of insulin, which indicates normal or low levels of glucose.
2. Yellow bubbles indicate moderate secretion of insulin, which indicates moderately elevated blood glucose levels.
3. Red bubbles indicate high insulin secretion, which indicates elevated glucose levels that require a strong metabolic response.

The speed and size of the bubbles increased as the glucose level rose, visually representing the increased release of insulin by the pancreas. This visualization helps patients and clinicians quickly interpret metabolic states and understand how lifestyle factors influence glucose regulation. The implementation was carried out in Python 3.12 using the TensorFlow/Keras 2.21.0, Scikit-learn 1.9.0, NumPy 2.5.2, Pandas 3.0.5, and Matplotlib libraries 3.11.1.

### 6.4. Performance Metrics

**Figure 7:**
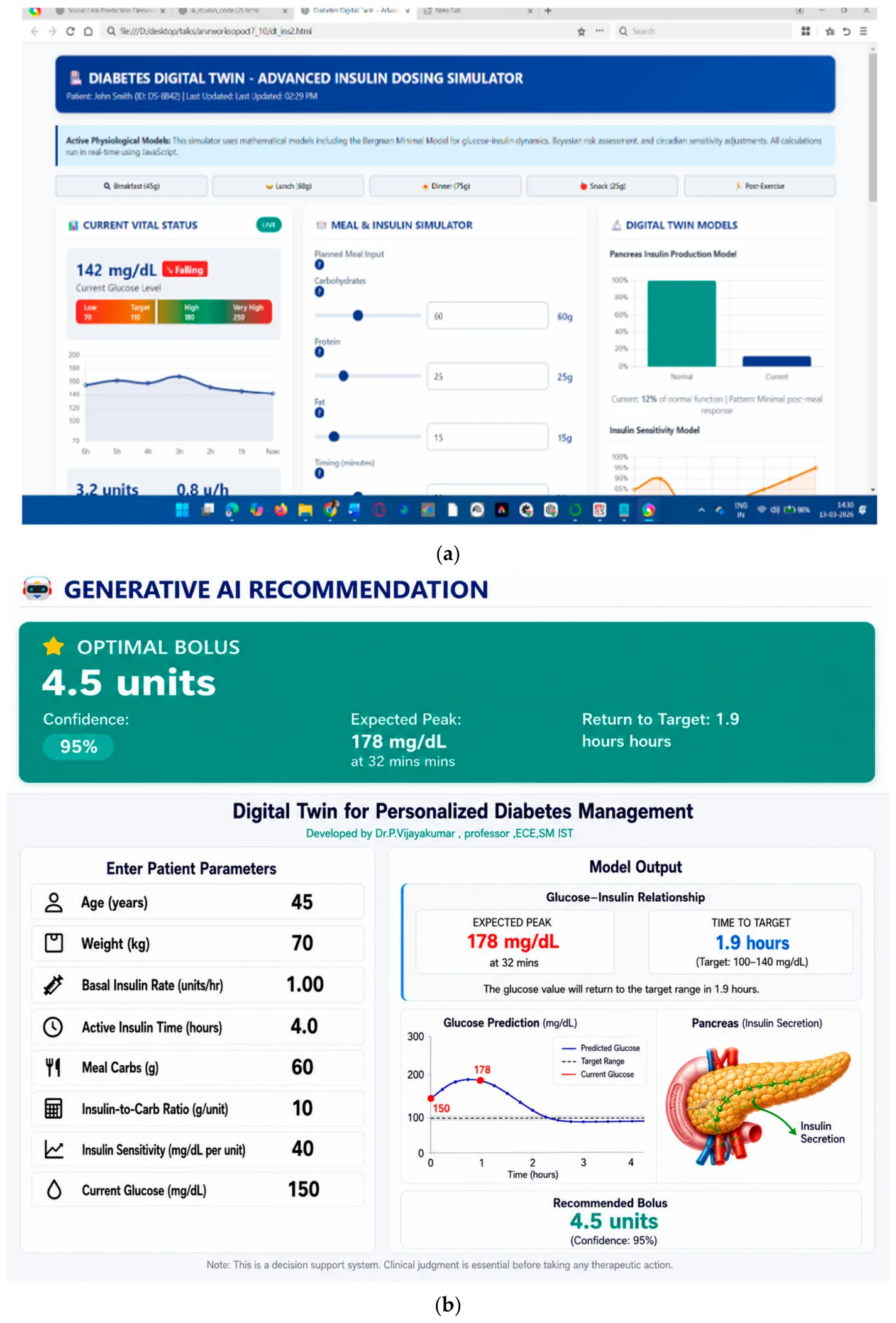
(**a**) Dashboard for real-time monitoring by DT and *(***b**) dashboard for DT generative AI recommendation.

System performance was evaluated using the following metrics. Figure 7a,b illustrate the data collection and dashboard for the proposed model.

#### 6.4.1. Prediction Accuracy

Prediction accuracy measures the closeness of the reference or truth values of glucose over time to the predicted values of glucose in the blood. Equations (17) and (18) provide the accuracy results.

#### 6.4.2. Glucose Stability

The stability represents the extent to which the glucose level was maintained by the system within a predefined safe limit range. It indicates the effectiveness of the control. Mathematical expressions (19)–(24) are used for stability evaluation.

#### 6.4.3. Hypoglycemia and Hypoglycemia Detection Rate

It quantifies how consistently and accurately the system detects the risks or episodes of low levels of glucose in the blood, which is critical for the safety of the patient. Step 10 provides a well-quantified analysis for the prediction. The simulation results were also consistent with this.

#### 6.4.4. Experimental Validation

Experimental validation was carried out using the following experimental settings: the monitoring duration was 24 h with a sampling interval of 6 min for the digital twin simulation and model validation. All outputs were successfully generated with the feature names in SHAP. The training ratio was 0.8, the testing ratio was 0.2, and cross-validation was carried out with five folds. The samples for training and testing were 240 and 60, respectively. The prediction horizon is one step ahead of the glucose prediction. Table 4 lists the significant results.

Using simulation, the training data with the proposed models were obtained and are presented in Table 5.

## 7. Results and Discussion

The proposed model of DT simulation results demonstrates that the model can reproduce realistic fluctuations in glucose, which are driven by meals, influenced by the circadian cycle, and influenced by physical activity. The following outcomes are obtained from the DT model: 1. Glucose prediction (post-meal):Both the magnitude and timing of glucose peaks are captured after carbohydrate intake. The system provides accurate predictions of postprandial glucose excursions. 2. Outcome of physical activity: During and after exercise sessions, glucose levels decrease, and this is effectively modelled with simulations. Physical activity impacts glucose reduction. 3. Significance of circadian modelling: When modelling glucose dynamics at different times of the day, incorporating circadian variations in insulin sensitivity will improve overall prediction and accuracy. 4. Detection of hyperglycemia at the early stages: Preventive measures can be adopted in the early stages by estimating glucose levels using the Bayesian risk estimation module. This estimation model will provide elevated levels of hyperglycemia in the early stages.

Compared with traditional physiological models, the proposed DT framework provides:

- Better control of glucose fluctuations.
- Guidance for more individualized insulin dosage, delivered at the right time.
- Improved identification of potential metabolic risks.

These outcomes demonstrate the advantages of the proposed method and underline its potential to support more precise and personalized diabetes care in the future.

**Figure 8:**
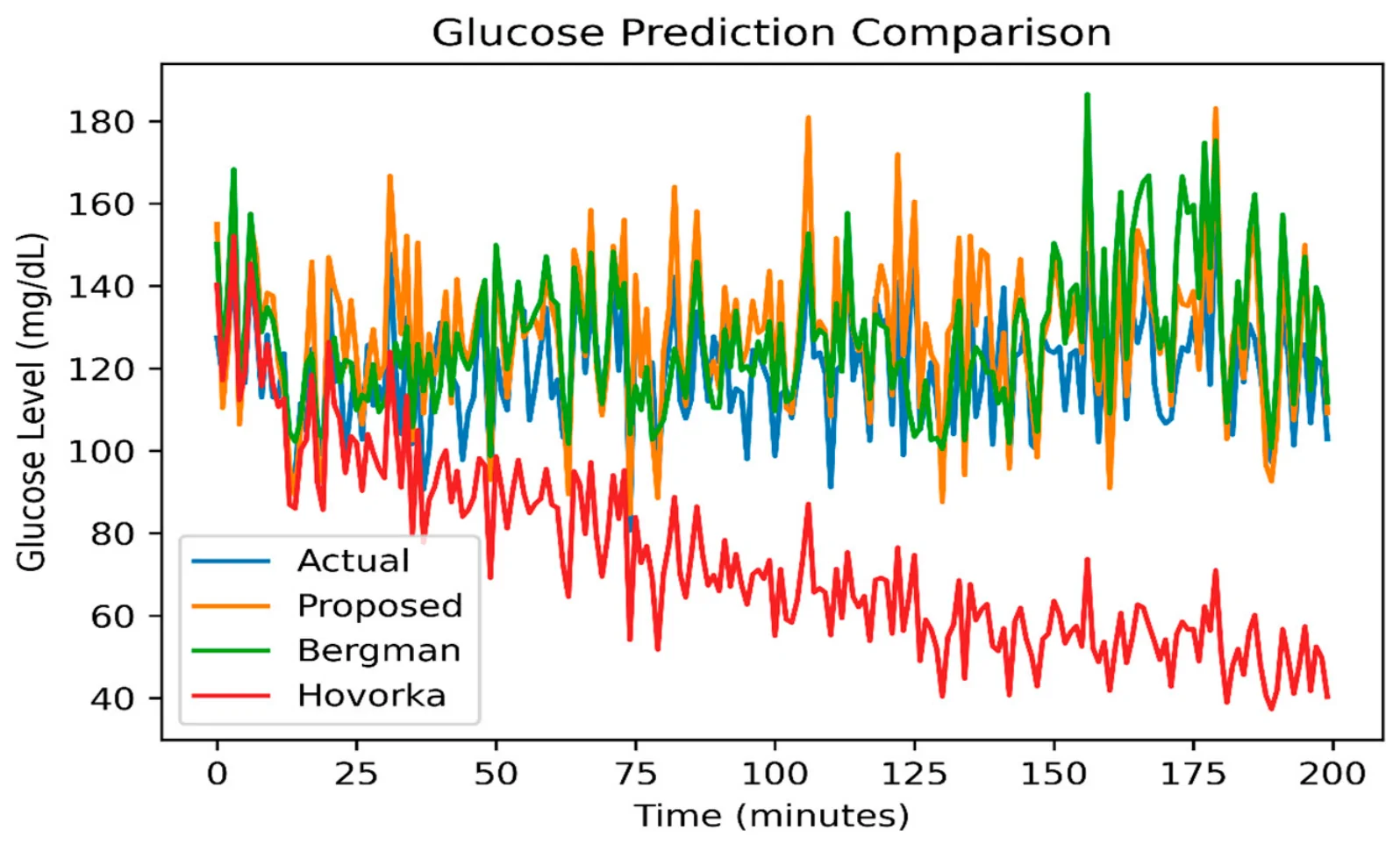
Comparison of glucose prediction.

**Figure 9:**
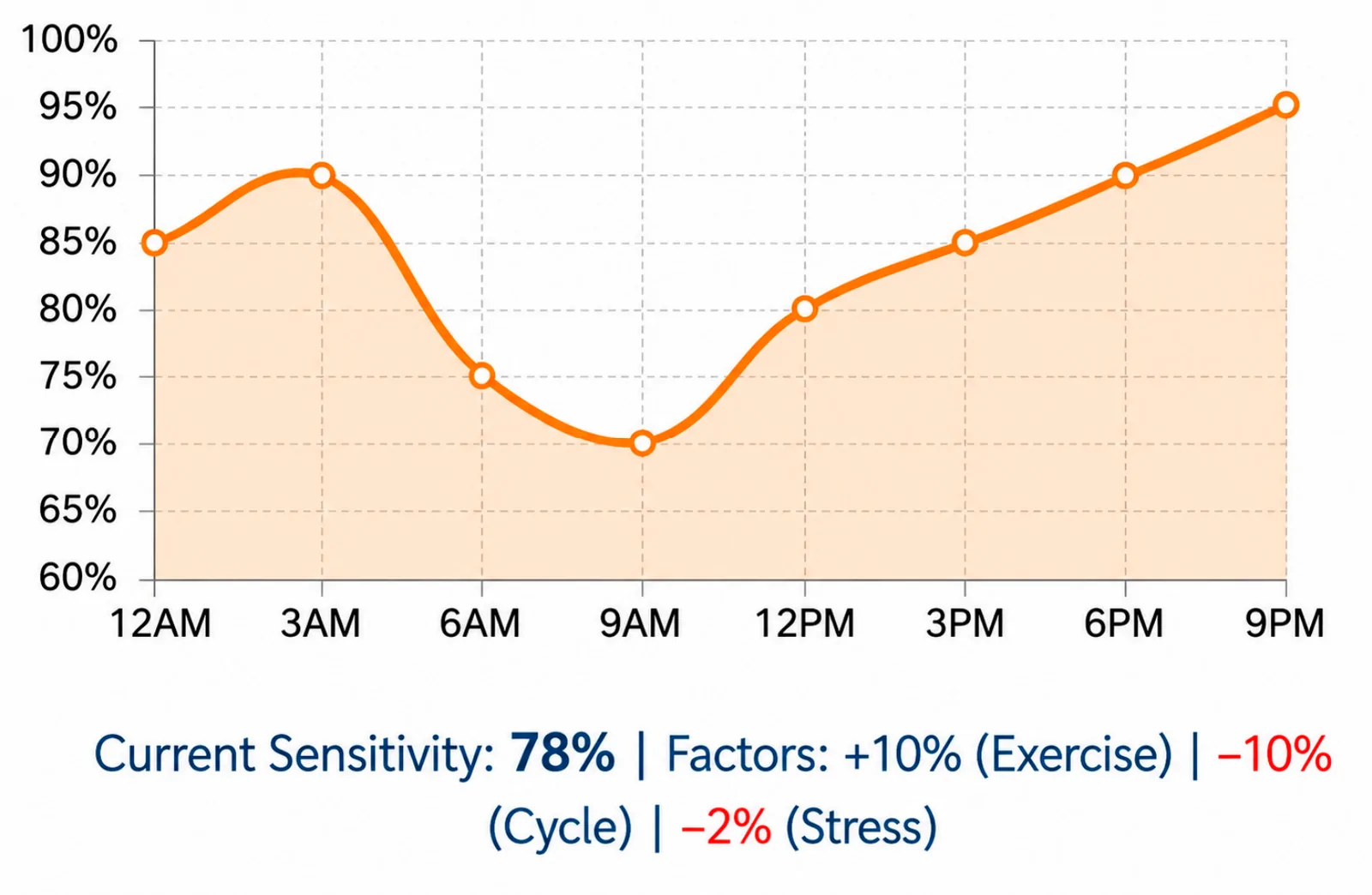
Insulin sensitivity models.

Figure 8 illustrates the comparative glucose prediction performance of the proposed DT model against the Bergman and Hovorka physiological models. The proposed hybrid model highlights the advantage in real-time monitoring of metabolic variability. The Bergman model exhibits delayed responsiveness with smoother dynamics. A more detailed physiological description with dynamic lifestyle variations is provided by the Hovorka model. The actual glucose trajectory, which captures short-term fluctuations and long-term variations, demonstrated closer alignment with the proposed combined model. Classical approaches do not consider lifestyle factors such as food intake, physical activity, and sleep. The integration of these factors is not provided by the existing models. However, the proposed model overcomes all the drawbacks of conventional models. Figure 9 shows the details of insulin sensitivity obtained by running the simulation.

**Figure 10:**
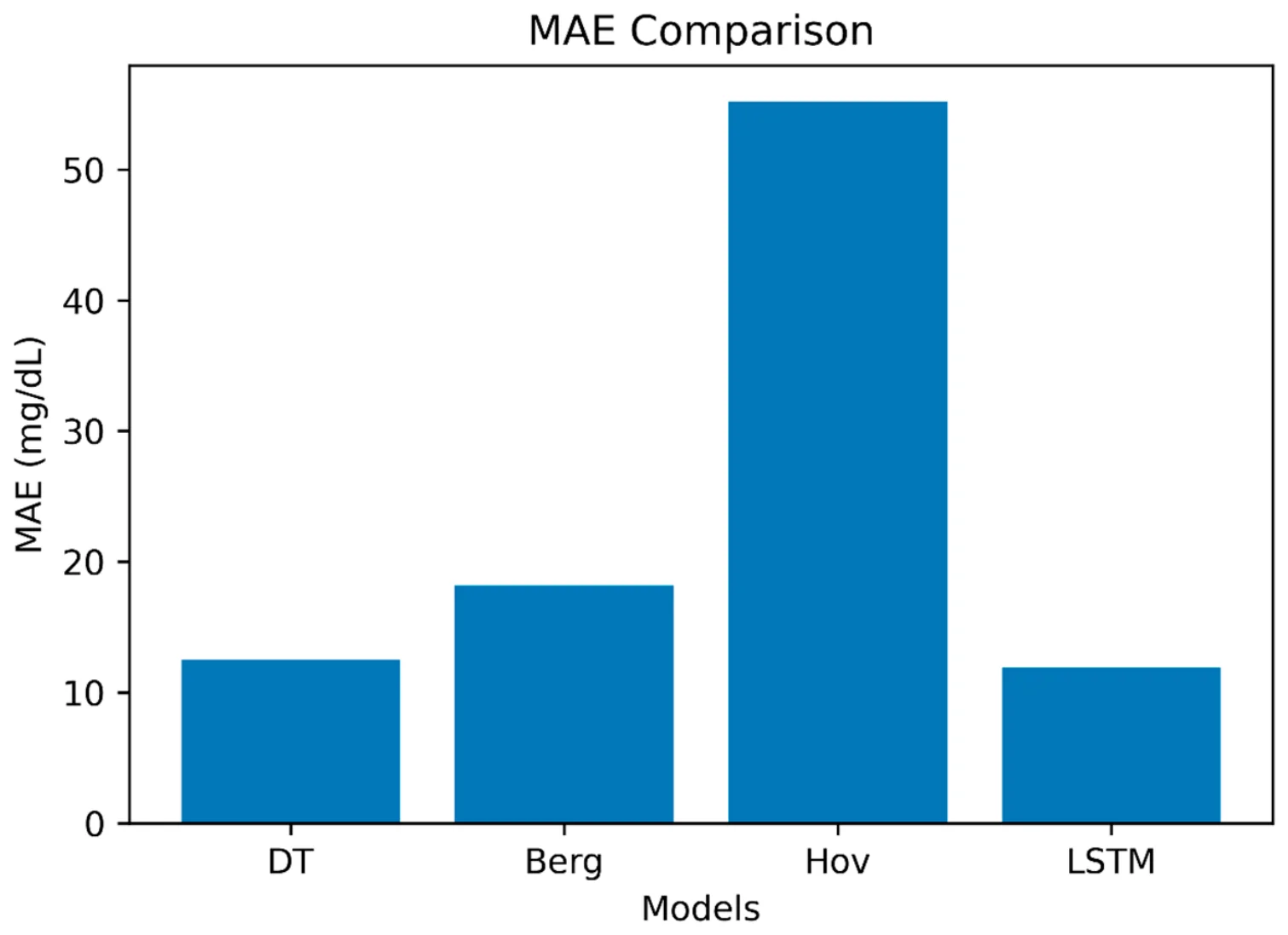
Comparison between models.

Figure 10 presents a comparison of the mean absolute error (MAE) across the models. The proposed hybrid model (Hovorka + LSTM) exhibits a lower MAE and provides superior pointwise prediction accuracy. The highest MAE values were due to the Bergman and Hovorka models, whereas the LSTM model exhibited competitive performance. In clinical scenarios, even small prediction errors can significantly affect insulin dosage.

**Figure 11:**
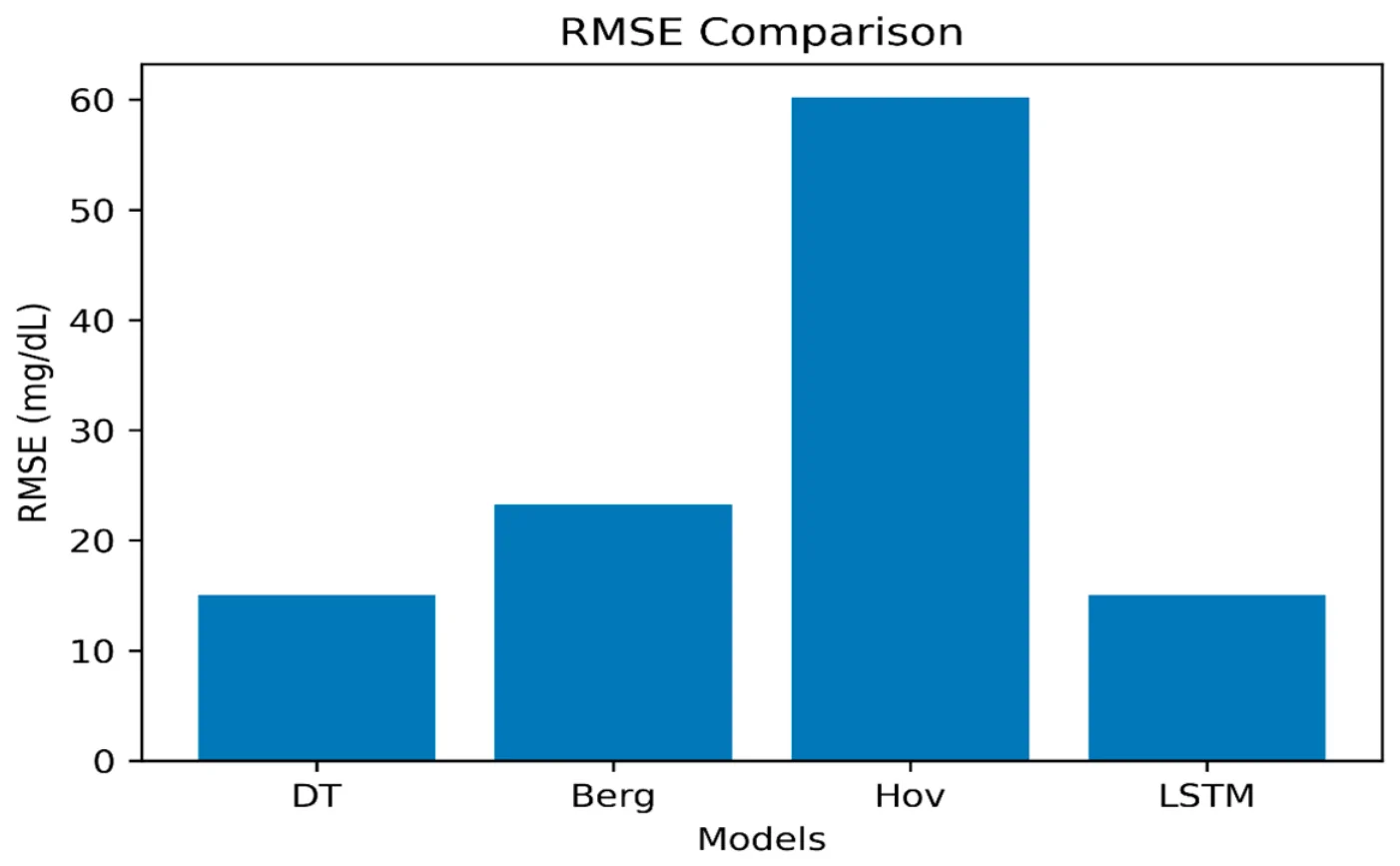
RMSE across models.

Figure 11 shows the root mean square errors (RMSEs) across the different models. The inability to capture the glucose variations provided by the Bergman and Hovorka models resulted in a higher RMSE. The proposed model outperformed the others with a minimum RMSE, ensuring stability and preventing acute complications of hyperglycemia and hypoglycemia.

**Figure 12:**
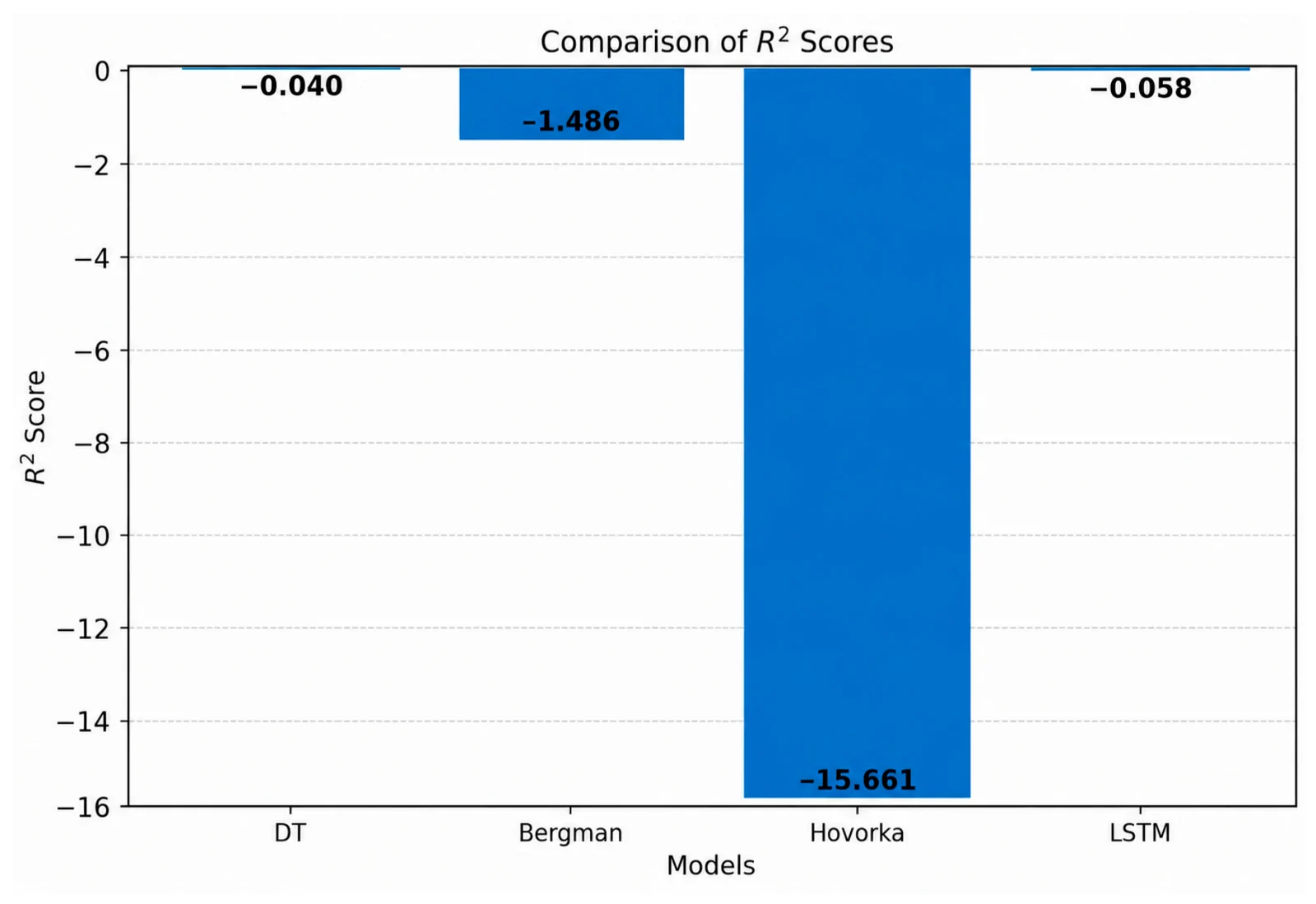
LSTM-based glucose prediction.

**Figure 13:**
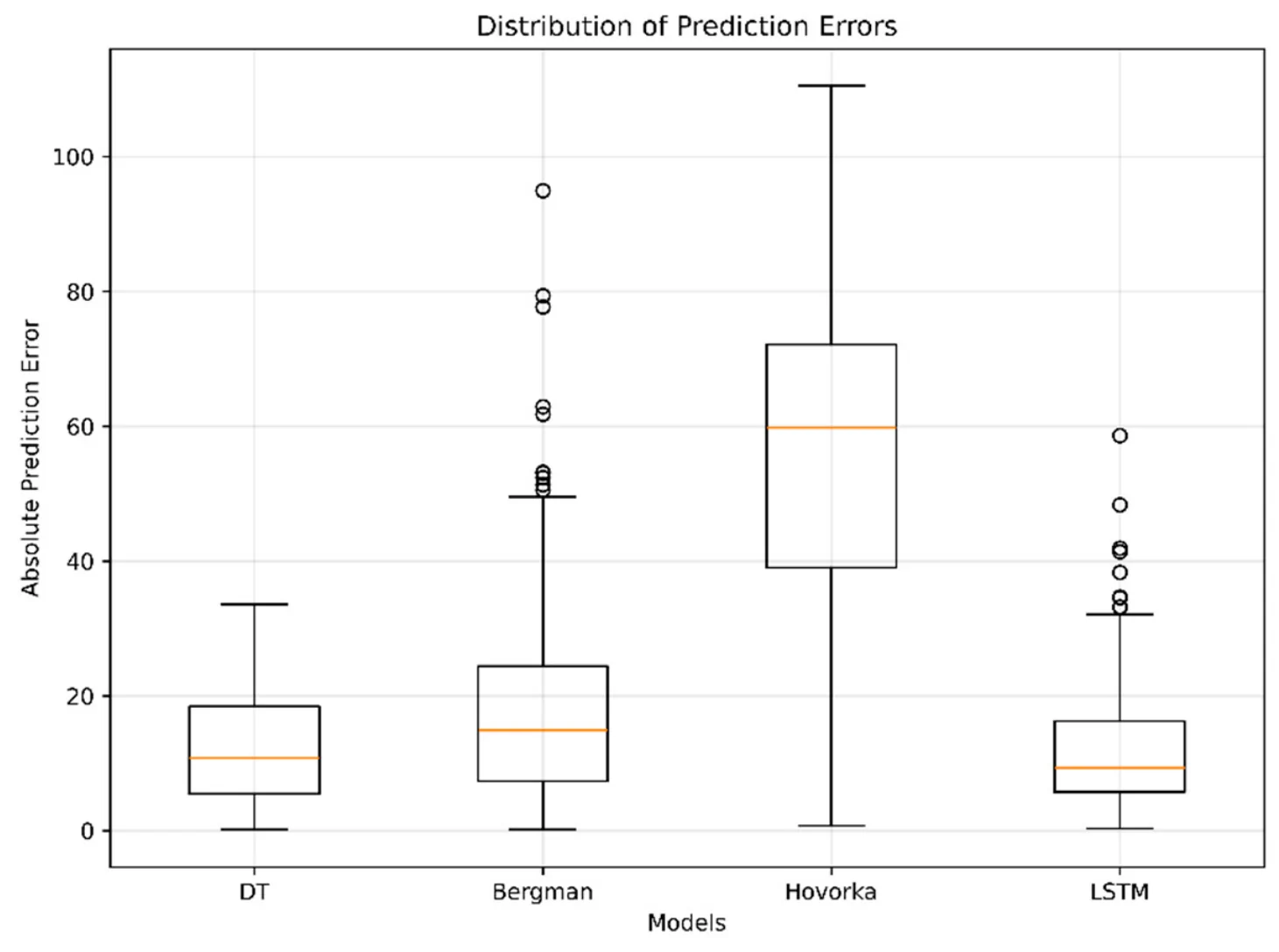
Paired *t*-Test.

Figure 12 shows LSTM-based glucose prediction compared with the actual measurements. The proposed DT model motivates the integration of XAI and physiological events in the prediction of glucose. Temporal and nonlinear patterns were observed in the LSTM model, along with a high predictive capability. A paired *t*-test was performed, and the results are shown in Figure 13, validating these results. A comprehensive statistical significance analysis was performed to validate the superiority of the proposed DT model. A paired *t*-test was performed to compare the prediction errors of the proposed model with those of each baseline model (Bergman, Hovorka, and LSTM).

The hyperparameters were selected using a grid search with a learning rate of 0.001, a batch size of 32, and 100 epochs. The model was trained using the Adam optimizer with the mean squared error (MSE) as the loss function. A 5-fold cross-validation strategy was employed to evaluate the model’s robustness and reduce overfitting. The paired *t*-test and one-way ANOVA results are shown in Table 6.

**Figure 14:**
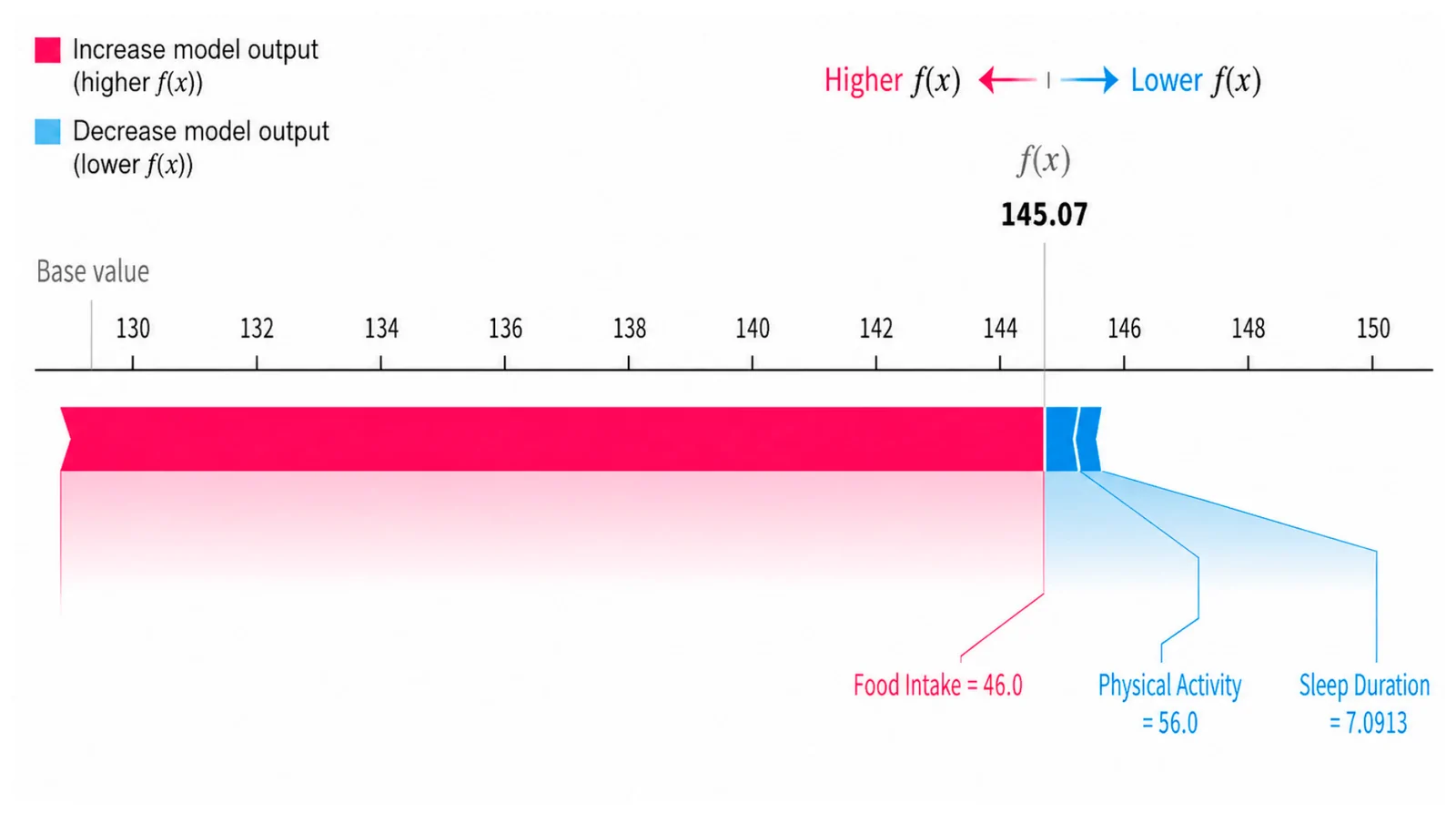
SHAP-based analysis results.

Figure 14’s positive SHAP values indicate an increase in glucose levels, whereas the negative values correspond to glucose reduction. By highlighting the contribution of each input variable to glucose prediction, the proposed DT model provides clinically interpretable insights, making it suitable for real-world deployment. To improve the interpretability of the proposed DT model, SHAP was employed using a representative background dataset of 200 samples selected from the training dataset. The SHAP ranking and force plots provide transparent explanations that can be readily interpreted by clinicians, enabling the identification of the most influential physiological factors affecting the predicted glucose level. The features of the XAI framework, including food intake, physical activity, sleep, medication, dietary intake, and glucose utilization, are listed in Table 7.

**Figure 15:**
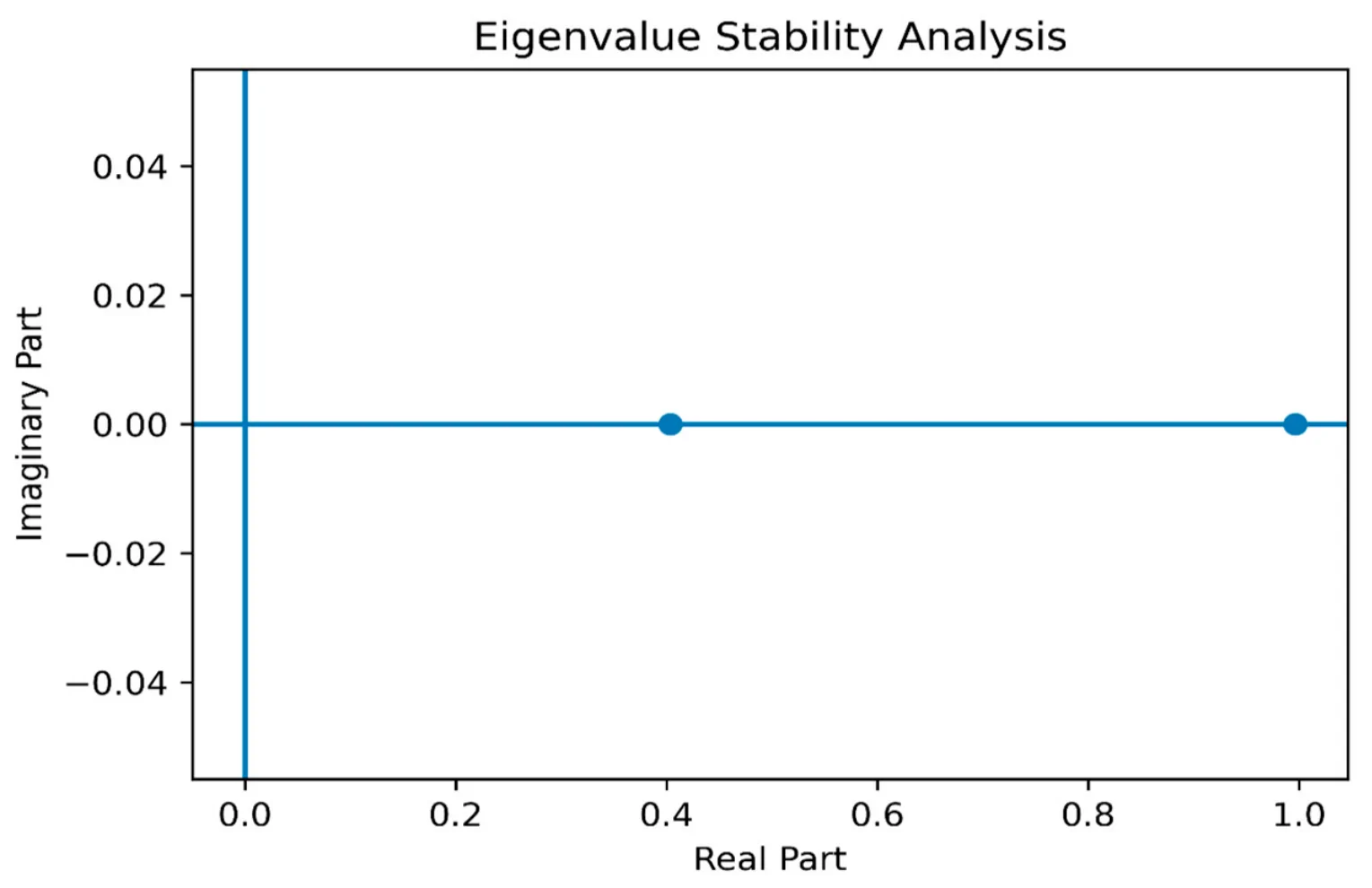
Eigenvalue-based stability analysis.

Figure 15 illustrates the eigenvalue-based stability analysis of the proposed DT system. Blue dots represent the eigenvalues of the system: $\lambda_{1}=0.4$, $\lambda_{2}=0.96$. The horizontal blue line at y = 0 represents the real axis, where the imaginary part of the eigenvalue is zero. The vertical blue line at x = 0 represents the imaginary axis, where the real part is zero. Because all eigenvalues are located on the real axis, the system has no oscillatory component (imaginary part = 0). For any discrete-time system, the criterion for the stability is the absolute value of eigenvalues to be less than one (∣λ∣ <1). Since our eigen values are less than 1 ($\lambda_{1}=0.4$, $\lambda_{2}=0.96$), indicating that our system was stable.

The Lyapunov-based validation further confirmed the asymptotic stability of the system, supporting its reliability for long-term glucose monitoring applications. Even small perturbations may contribute to the critical stability factor. The system is stabilized if the eigenvalues are bounded by the region of convergence. All the eigenvalues of the system matrix were strictly negative, indicating asymptotic stability. Furthermore, the Lyapunov matrix P v obtained from the continuous Lyapunov equation is positive definite, confirming the global asymptotic stability of the closed-loop DT. The values are listed in Table 8.

Table 9 summarizes the quantitative performance metrics of all the models. The proposed DT model achieved the best performance across the RMSE, MAE, and R^2^ metrics compared to the conventional approaches. The hybrid model integrates both mathematical and AI models for superior performance.

Ethical clearance: The following steps were taken to maintain the standard health protocol, and a description of the collected data characteristics is as follows:

- Ethical clearance was obtained from the SRM Medical College Hospital and Research Centre (ethical clearance number: 8274/IEC/2022).
- Relevant guidelines and regulations were followed for all methods.
- All experimental protocols were approved by the SRM Medical College Hospital and Research Centre.
- A doctor from SRM Medical College Hospital and Research Centre was involved in the current study.
- Informed consent was obtained from each volunteer.

The volunteer’s finger was cleaned with isopropyl alcohol before reading the invasive sample. A new set of lancets and strips was used for each sample. Data collection was performed according to the steps described in Section 6.3.

## 8. Conclusions

The proposed DT system framework combines physiological modelling, lifestyle factor analysis, predictive components, probabilistic risk assessment, and interpretability features within a single integrated framework. Simulation results showed that the approach provides more accurate glucose predictions, improved identification of glycemic risk conditions, and more tailored insulin dosing than conventional methods. These findings suggest that the DT approach can serve as an effective tool for clinical decision support and personalized treatment planning in diabetes management.

Future work will involve connecting the framework with real-time continuous glucose monitoring systems, validating its performance using clinical datasets, and further enhancing its personalization and interpretability for practical use in the healthcare sector. In future work, automation needs to be incorporated into the DT-assisted framework for personalized medicine. Cloud-based implementation and standardized interoperability protocols can further enhance the scalability of large-scale clinical use. In the future, federated learning and edge computing may also be adopted to minimize the sharing of raw patient data. The dependence on high-quality sensor data and uninterrupted connectivity may also affect the system performance. In addition, physiological variability, sensor noise, and missing data can reduce the prediction accuracy. Future work will focus on developing computationally efficient algorithms, lightweight models, and optimized edge/cloud computing solutions to improve the real-time performance while reducing the operational costs. Future work involving large-scale multicenter clinical validation and optimization for resource-efficient deployment is also discussed.

## Figures and Tables

**Table 1:** Comparison of the different ML models.

Model	Key Features	Advantages	Limitations
Bergman Minimal Model [10]	Simple differential equation-based glucose–insulin model	Low complexity, easy implementation	No lifestyle factor integration
Hovorka Model [16]	Detailed physiological glucose–insulin dynamics	High physiological accuracy	Complex parameter calibration
Long Short-Term Memory Neural Network [17]	Deep learning model for CGM time-series prediction	Accurate glucose trend prediction	Requires large datasets, low interpretability
Hybrid Model (Hovorka + LSTM)-Combined Model	Combines physiological and AI-based prediction	Improved personalization and prediction accuracy	Higher computational complexity

**Table 2:** Novelty of the proposed model.

Sl. No.	Research Contribution	Description	Benefit of This Research Work
1	Hybrid DT framework	Combines mechanistic physiological models with data-driven analytics. Uses more personalized simulation of glucose–insulin dynamics	Improved accuracy in personalized medicine
2	Circadian Modelling	Long-term prediction accuracy is improved	Metabolic fluctuations are predicted to enhance performance behaviour
3	Probabilistic Risk Assessment	Hyperglycemicand hypoglycemic predicted in a probabilistic manner	Probabilistic risk forecasting is performed
4	Explainable AI Methods	Better clinical support to patients	Transparent decision support system for clinicians and patients

**Table 3:** (**a**) Details of the niGLUC-2.0v data samples and (**b**) demographic characteristics of the participants.

**(a)**		
**niGLUC-2.0v**	**Sample Data from Finger**	**Sample Data from Wrist**
Diabetic samples	Male (M): 107	Male (M): 107
	Female (F): 85	Female (F): 85
Age in groups (years)		
(20–35)	M: 6, F: 0	M: 6, F: 0
(35–50)	M: 22, F: 16	M: 22, F: 16
(50–65)	M: 67, F: 53	M: 67, F: 53
(65–90)	M: 12, F: 16	M: 12, F: 16
Prediabetic samples	Male (M): 6	Male (M): 6
	Female (F): 2	Female (F): 2
Age in groups (years)		
(20–35)	M: 2, F: 0	M: 2, F: 0
(35–50)	M: 4, F: 2	M: 4, F: 2
(50–65)	M: 0, F: 0	M: 0, F: 0
(65–90)	M: 0, F: 0	M: 0, F: 0
Repeated samples	Male (M): 6	Male (M): 6
	Female (F): 2	Female (F): 2
(50–65)	M: 2, F: 2	M: 2, F: 2
(65–90)	M: 4, F: 0	M: 4, F: 0
Total samples	Male (M): 113	Male (M): 113
	Female (F): 88	Female (F): 88
Total samples	201	201
**(b)**		
**Characteristics**	**Value**	
Total participants/Volunteers	201	
Mean age (SD)	57 ± 11 years	
Age range	25–78 years	
Age groups	20–35 years: 8 (4%)	
	35–50 years: 44 (21.9%)	
	50–65 years: 121 (60.2%)	
	65–90 years: 28 (13.9%)	
	Male: 113 (56.2%)	
	Female: 88 (43.8%)	
BMI(kg/m^2^) ranges	<18.5	
	18.5–24.9	
	25.0–29.9	
	>30.0	

**Table 4:** Experimental results.

No. of Folds	RMSE	MAE	R^2^
1	15.83	13.417	0.675
2	14.729	12.297	0.156
3	14.628	12.011	0.04
4	15.707	12.776	0.042
5	14.015	11.833	0.031
Average	14.982 ± 0.688	12.467 ± 0.572	0.094 ± 0.297

**Table 5:** Training data with the proposed models.

Model	Training RMSE	Training MAE	Training R^2^	Testing RMSE	Testing MAE	Testing R^2^	Prediction Time (s)
Digital Twin	15.04146	12.51267	−0.03955	15.04146	12.51267	−0.03955	0.003364
Bergman	23.26219	18.19376	−1.48637	23.26219	18.19376	−1.48637	0.001384
Hovorka	60.21732	55.19903	−15.6612	60.21732	55.19903	−15.6612	0.001313
LSTM	15.04666	11.9384	−0.03189	15.04666	11.9384	−0.03189	0.338411

**Table 6:** Paired *t*-test and one-way ANOVA.

Sl. No.	Parameters	DT vs. Bergman	DT vs. Hovorka	DT vs. LSTM
1	t-statistic	−6.7075	−27.8032	0.6746
2	*p*-value	9.94 × 10^−11^	8.98 × 10^−85^	0.50045828
3	Inference	Statistically Significant	Statistically Significant	Not Significant
4	**One-Way ANOVA**			
	F-statistic 506.6025397639994			
	*p*-value 4.284068402531422 × 10^−210^	There is a significant difference among the models.		

**Table 7:** Clinical interpretation of the results.

Feature	Clinical Interpretation	Mean |SHAP|
Food Intake	Higher food intake increases predicted glucose level	10.34688
Physical Activity	Increased activity reduces glucose concentration	1.39
Sleep Duration	Longer sleep improves glucose regulation	0.304660697
Medication	Medication assists glucose stabilization	0

**Table 8:** Eigenvalues from the simulation.

Eigenvalues	Lyapunov Matrix P	Eigenvalues of P
(−0.47278537346672106 + 0j)	[0.98616978 0.16010356]	[1.06421879 0.65774605]
(−0.9572146265332789 + 0j)	[0.16010356 0.73579507]	Positive Definite P

**Table 9:** Comparison report of different models from the simulation.

Model	RMSE (mg/dL)	MAE (mg/dL)	RÂ^2^ Score
Proposed DT	15.04146	12.51267	−0.039545527
Bergman	23.26219	18.19376	−1.486365974
Hovorka	60.21732	55.19903	−15.66121107
LSTM	15.11142	11.89696	−0.040789409

## Data Availability

The data presented in this study are available on request from the corresponding author due to privacy and ethical reason.
